# Aortic valve replacement and repair of left ventricular pseudoaneurysm in a Jehovah’s Witness

**DOI:** 10.5812/cardiovascmed.7274

**Published:** 2013-02-24

**Authors:** Andrea Perrotti, Claude Vaislic, Sidney Chocron

**Affiliations:** 1 Department of Thoracic and Cardiovascular Surgery, EA3920, University Hospital Jean Minjoz, 25000, Besançon, France; 2 Centre Medico-chirurgical Parly II, 21 rue Moxouris, 78150, Le Chesnay, France

**Keywords:** Jehovah's Witnesses, Left Ventricular, Aneurysm, False

## Abstract

The preoperative and surgical management of a giant left ventricular pseudoaneurysm(LVP) associated with aortic valve replacement in a 76 year old male Jehovah’s Witness patient is reported. The satisfactory recovery observed in this patient demonstrates the feasibility of this complex surgical procedure even in this particular patient population.

## 1. Introduction

Due to their faith, Jehovah’s Witness patients refuse any transfusion of blood or blood products, i.e. plasma or platelets. This refusal increases the risk of death and makes cardiac operations challenging in these patients. Here is described the management and modalities of operation in a Jehovah’s Witness patient who had a giant post-infarction left ventricular pseudoaneurysm associated with aortic stenosis and a stenosed left ventricular descending artery.

## 2. History

**Figure 1.**
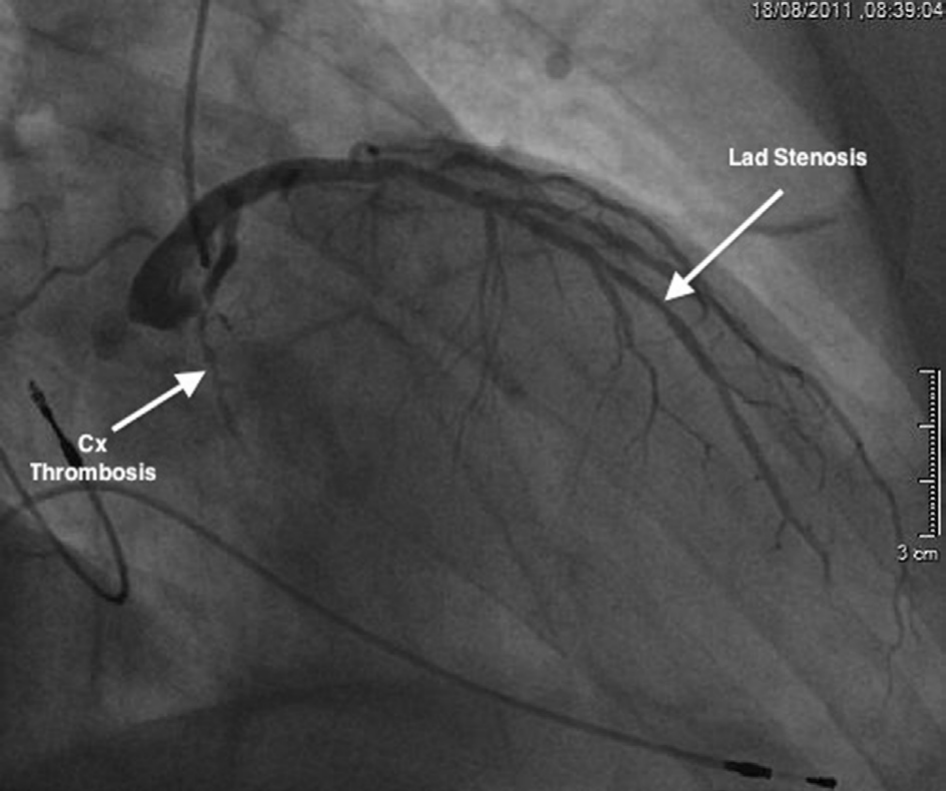
Coronary angiography showing the 60% left anterior descending artery (LAD) stenosis and the circumflex artery (CX) thrombosis

**Figure 2.**
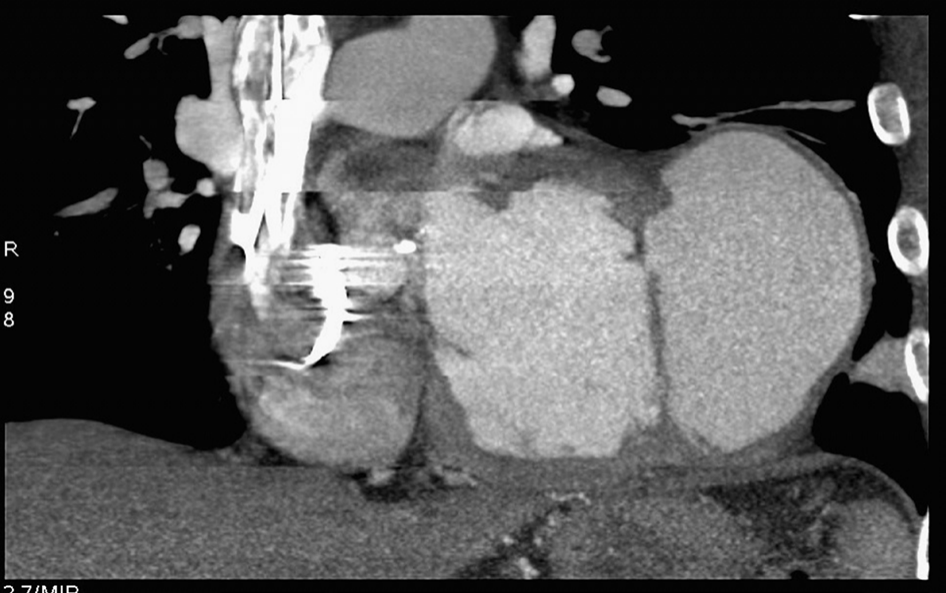
Preoperative CT scan showing the left ventricular pseudoaneurysm

**Figure 3.**
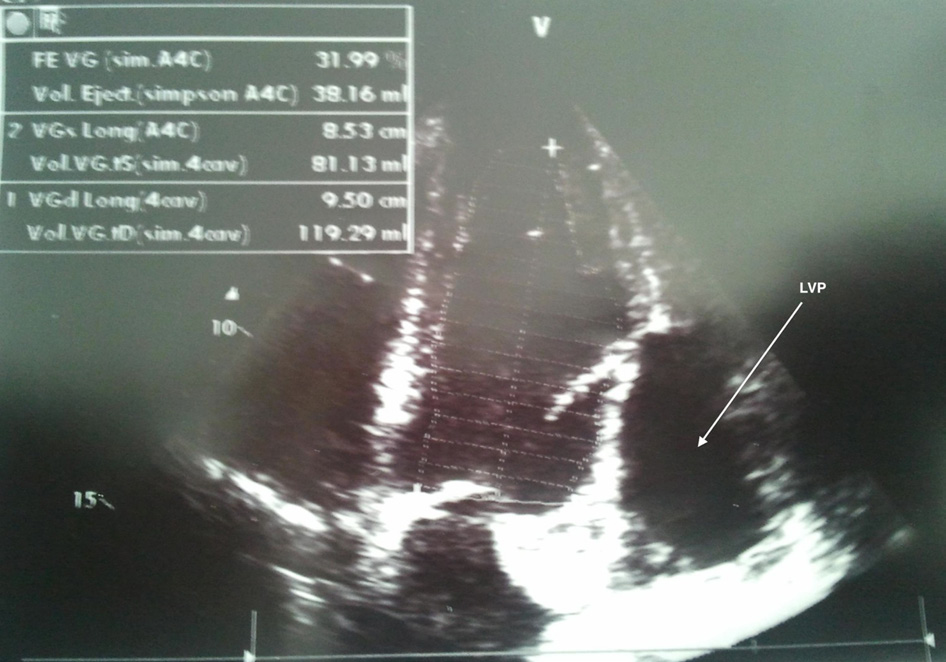
Echocardiography view showing the left ventricular pseudoaneurysm (LVP)

A 76 year-old Jehovah’s Witness patient was admitted to our institution in July 2011 with postero-lateral myocardial infarction (MI). Risk factors were hypertention and hypercholesterolemia. Coronary angiography revealed an occlusion of the circumflex artery with a 60% left anterior descending artery (LAD) stenosis (Figure 1). Myocardial perfusion scintigraphy was planned to decide on the need for angioplasty of the LAD, but was not performed. Six months later, the patient was re-admitted with dyspnea and chest pain. CT scan (Figure 2) and subsequent echocardiography (Figure 3), showed a giant left ventricular pseudo-aneurysm (10 cm x 10 cm x 5 cm), partially thrombosed, and likely caused by a contained lateral free wall rupture secondary to the initial MI. In addition, echocardiography showed a calcified stenosis of the aortic valve with a mean transvalvular gradient of 50 mmHg. The left ventricular ejection fraction was 40%. It was decided to perform exclusion of the false aneurysm associated with aortic valve replacement. In order to minimize the risk of post-operative bleeding, coronary artery bypass graft surgery on the LAD was not performed, and it was decided to treat it at a later date by coronary stenting, depending on the result of postoperative myocardial perfusion scintigraphy. 

## 3. Preparation of the Patient

The patient was informed that, in line with his wishes, the operation could not be performed in an emergency, and that he presented an increased risk of death while awaiting operation. At admission, the patient’s hemoglobin concentration was 13.3 g/dL. In the institution, it is necessary for the patient to have a hemoglobin concentration of at least 14 g/dL in order to avoid transfusion. To reach this goal, the Cornell University Protocol was used (1, 2). Injections of human erythropoietin were administered at a dose of 40,000 IU subcutaneously every 3 days, associated with ferrous fumarate at a dose of 1 g, 3 times a day. The goal of 14 g/L haemoglobin was reached after 30 days of treatment.

## 4. Surgery

**Figure 4.**
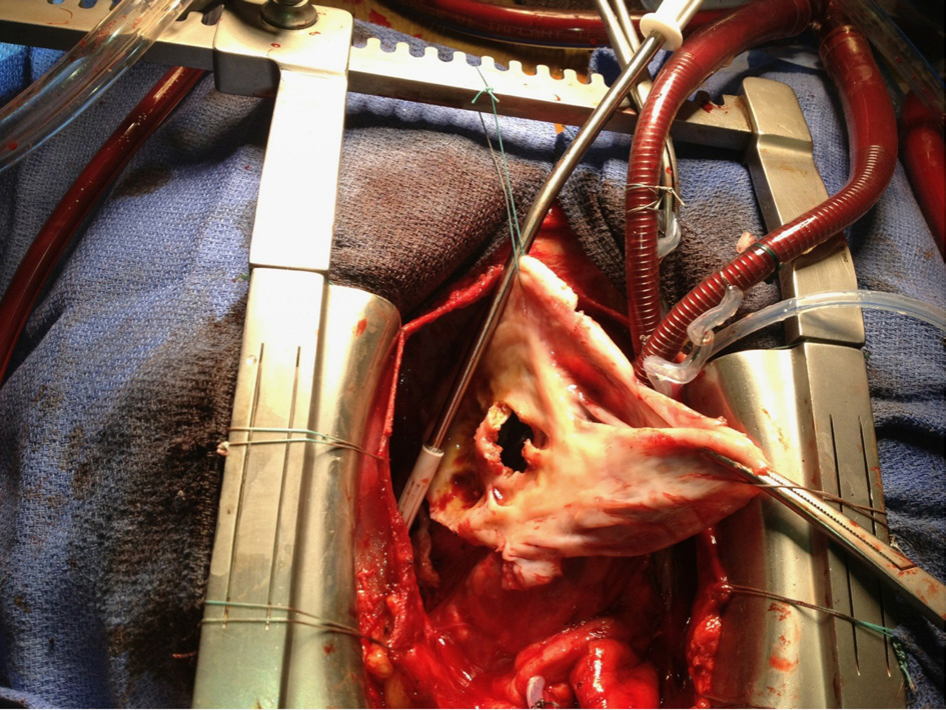
Operative view. The left ventricular pseudoaneurysm is opened showing the communication between the left ventricle and the false aneurysm

An antifibrinolytic agent (tranexamic acid, 1 g) was injected at induction and after the cardiopulmonary bypass (CPB) weaning. The operation was carried out through a median sternotomy. Intra-operative blood salvage was performed using CellSaver® Elite® Autotransfusion System (Haemonetics Corporation, Braintree, MA, USA). Cardiopulmonary bypass was performed by mini-extracorporeal circulation (MECC)with retro-priming using the patient’s blood. Hemoglobin concentration during CPB was 10.5 g/dL. Because of gross adhesions and hemodynamic instability first a partial dissection limited to the ascending aorta and right atrium was prformed. The cardiopulmonary bypass was established between the ascending aorta and the two vena cava. The left ventricle was vented through the aorta and the right superior pulmonary vein. Because of the danger of systemic embolisation, the dissection was completed only after the aorta was cross-clamped. Myocardial protection was provided by intermittent cold anterograde cardioplegia, first through the aortic root, and subsequently directly in the coronary ostia. Moderate hypothermia (32°C) was used. The aneurysm was opened, the thrombus removed, and the orifice of communication closed using a Dacron patch sutured with polypropylene sutures (Figure 4). Tissucol® (Baxter International Inc., Deerfield, IL, USA) was used to achieve better hemostasis of the repair. Then, aortic valve replacement was performed using a 23 mm Trifecta® Valve (St. Jude Medical, Inc., St. Paul, MN USA). The aortic cross-clamp time was 86 minutes and the total CPB lasted 111 minutes. Meticulous attention was paid to hemostasis. The patient made a good recovery and was discharged to home on postoperative day 9 with a hemoglobin concentration of 10.3g/dl. The patient was discharged with the following treatment : warfarin, aspirin, beta-blocker, and furosemide.

## 5. Discussion

Despite the high mortality reported in large series (1), surgical treatment of pseudoaneurysm of the left ventricle is mandatory when the size is large, or when it increases quickly. Other compelling reasons to operate on these patients are that firstly, there exists a risk of embolization of thrombotic material, and secondly, the patient usually presents symptoms like chest pain or dyspnea (2). This complex operation was rendered particularly challenging in the case reported here, due to the fact that the patient refused blood transfusion on religious grounds. Repair of a giant left ventricular pseudoaneurysm in a Jehovah’s Witness patient has already been performed (3). However, in this specific case, repair was associated with aortic valve replacement. The bypass on the LAD was not performed, since PCI was chosen as an alternative treatment approach that would reduce the risk of postoperative bleeding. In fact, postoperative myocardial perfusion scintigraphy performed 4 months after the operation showed no apical defect. Therefore, the complementary angioplasty was not performed. A recent review by Jassar et al. (4)showed that mortality of Jehovah’s Witness patients is not higher if the patient is stabilized before surgery. The preoperative use of erythropoietin and high doses of iron fumarate is indicated to stimulate erythropoiesis and increase hematocrit to obtain a minimum hemoglobin level of 14g/dL. The patient has to be informed of the risk of death during the time awaiting surgery. Discontinuation of aspirin and clopidogrel before the operation is also mandatory. In this case, median sternotomy was the mandatory approach, but any approach that permits easy and bloodless dissection should be preferred. In order to minimize hemodilution, after cannulation of the ascending aorta and right atria, the priming of the bypass circuit was removed and replaced by retro-priming with the patient’s blood (5-7). Meticulous hemostasis is mandatory. In case of massive blood loss, the CBP is either not discontinued or restarted in order to recuperate the blood loss. The use of hemostatic glue, cryoprecipitate and factor VII may help the surgeon to improve hemostasis (4). Careful management of crystalloid fluid administration is necessary to prevent hemodilution. This case shows that, although technically demanding, good results can be achieved in Jehovah’s Witness patients undergoing complex surgery.
